# Retro analog concept: comparative study on physico-chemical and biological properties of selected antimicrobial peptides

**DOI:** 10.1007/s00726-017-2473-7

**Published:** 2017-07-29

**Authors:** Damian Neubauer, Maciej Jaśkiewicz, Dorian Migoń, Marta Bauer, Karol Sikora, Emilia Sikorska, Elżbieta Kamysz, Wojciech Kamysz

**Affiliations:** 1 Faculty of Pharmacy, Medical University of Gdańsk, Gdańsk, Poland; 2 Faculty of Chemistry, University of Gdańsk, Gdańsk, Poland

**Keywords:** Retro analog, Antimicrobial peptides, Omiganan, Pexiganan, CD experiments, Peptide hydrophobicity

## Abstract

Increasing drug resistance of common pathogens urgently needs discovery of new effective molecules. Antimicrobial peptides are believed to be one of the possible solutions of this problem. One of the approaches for improvement of biological properties is reversion of the sequence (retro analog concept). This research is based on investigation of antimicrobial activity against Gram-positive, Gram-negative bacteria, and fungi, hemolysis of erythrocytes, interpretation of the circular dichroism spectra, measurement of counter-ion content, and assessment of the peptide hydrophobicity and self-assembly using reversed-phase chromatography. The experiments were conducted using the following peptides: aurein 1.2, CAMEL, citropin 1.1, omiganan, pexiganan, temporin A, and their retro analogs. Among the compounds studied, only retro omiganan showed an enhanced antimicrobial and a slightly increased hemolytic activity as compared to parent molecule. Moreover, retro pexiganan exhibited high activity towards *Klebsiella pneumoniae*, whereas pexiganan was in general more or equally active against the rest of tested microorganisms. Furthermore, the determined activity was closely related to the peptide hydrophobicity. In general, the reduced hemolytic activity correlates with lower antimicrobial activity. The tendency to self-association and helicity fraction in SDS seems to be correlated. The normalized RP-HPLC—temperature profiles of citropin 1.1 and aurein 1.2, revealed an enhanced tendency to self-association than that of their retro analogs.

## Introduction

As a result of the widespread use of antibiotics, the appearance of infections caused by resistant organisms is on the rise. According to the reports of World Health Organization (WHO), the issue of antibiotic resistance is no longer the prediction for the future but it is the real problem nowadays (World Health Organization 2014). Since the treatment of common infections in the community and hospitals is being more difficult, patients remain infectious for a longer time and the healthcare costs rise. Acquisition of the non-susceptibility to antibiotics is also related to the circulation of genes in the environment and resistance determinants can be easily transferred to pathogenic microorganisms. The spread of these pathogens has been related to various epidemiological factors such as the lack of appropriate procedures in the healthcare facilities and the international transfer of patients coming from endemic areas (e.g., spread of carbapenem-resistant *Klebsiella pneumoniae* in Europe) (Girmenia et al. 2016). Increasing rate of multi-resistant bacteria forces the need of development of new antimicrobial substances that could be applicable in therapy.

Antimicrobial peptides (AMPs) seem to be an alternative to conventional antibiotics. Several peptides and peptide-based compounds that can be potentially used in the treatment of infectious diseases are passing clinical trials. Moreover, compounds from this category next to candidates for oncological treatment are reaching the top of therapeutic areas for peptides in Phase III of clinical pipeline (Kaspar and Reichert 2013). AMPs are compounds widely distributed in nature as they act as a part of innate immunity of organisms (Mansour et al. 2014). These compounds have attracted much attention as they are targeting broad-spectrum pathogens including bacteria, fungi, protozoa, and viruses (Zasloff 2002). Interestingly, they can also trigger and coordinate multiple components of innate and immune adaptive systems (Kamysz et al. 2003; Boman 2003; Schauber and Gallo 2007). Naturally occurring AMPs can vary in size, in majority having no more than 50 amino acids in sequence. Generally, these compounds contain about 50% of hydrophobic amino acids and simultaneously have a high content of basic amino acid residues such as histidine, lysine, and arginine (Hancock and Lehrer 1998). Such structure enables targeting the negatively charged surface of microorganisms.

Amphibian skin is a rich source of AMPs (Rinaldi 2002). Aurein 1.2 is an amphipathic and α-helical peptide originally isolated from Australian bell frogs, *Ranoidea aurea* (formerly *Litoria aurea*) and *Ranoidea raniformis* (formerly *L. raniformis*) (Rozek et al. 2000a, b). It is the smallest (13 residues) and the most extensively studied peptide from the group of aureins, owing to its antimicrobial and anticancer properties exhibited in vitro (Dennison et al. 2007; Giacometti et al. 2007; Lorenzón et al. 2014). Citropin 1.1 is a small antimicrobial peptide (16 residues) produced by dorsal and submental glands of the green tree frog, *Ranoidea citropa* (formerly *Litoria citropa*) (Wegener et al. 1999). It has been studied for its wide antimicrobial, anticancer and even nitric oxide synthase inhibitory activities (Wabnitz et al. 1999; Apponyi et al. 2004; Giacometti et al. 2005a, b). It is speculated that the mode of action of citropin 1.1 and aurein 1.2 is based on prerequisite aggregation and carpet-like mechanism since they both are too short to directly traverse lipid bilayer (Ambroggio et al. 2005). Another group of AMPs isolated from amphibians are temporins. These peptides were primarily isolated from the skin of the European red frog, *Rana temporaria*. The structure of temporins is highly variable and the majority of them contain a single basic residue (arginine or lysine). Temporin A is a well-known peptide from this group. It is a small (13 residues) molecule exhibiting antimicrobial properties (Simmaco et al. 1996). One of the characteristic features of AMPs is that only a part of their sequence may be critical for the antimicrobial activity. As a result, on the basis of the naturally occurring peptides, novel compounds of desirable properties could be synthesized. Thanks to those properties, many AMPs have been discovered. CAMEL or CA(1–7)M(2-9)NH_2_ is an example of a hybrid peptide (15 residues) containing fragments of two peptides with different antimicrobial activities (Andreu et al. 1992). First seven amino acid residues are derived from the sequence of cecropin A, a peptide isolated from the larvae of silk moth, *Hyalophora cecropia*. Other eight amino acid residues are derived from the sequence of melittin, the principal active component of apitoxin (honey bee venom) (Oh et al. 2000). This compound has been extensively studied for its broad-spectrum antimicrobial and anticancer activities (Smolarczyk et al. 2010). Omiganan (formerly MBI 226) is a small (12 residues) cationic peptide analog of indolicidin, which was originally isolated from the cytoplasmic granules of bovine neutrophils (Sader et al. 2004). Currently, omiganan pentachloride gel preparations undergo clinical trials in the treatment of *Rosacea* (Phase III), *Acne vulgaris*, genital warts and vulvar intraepithelial neoplasia (Phase II). Previously, it was also in Phase III clinical trials for the prevention of catheter-related bloodstream infections. Pexiganan (MSI-78) is an analog (22 residues) of a magainin, a peptide originally isolated from the African clawed frog, *Xenopus laevis* (Ge et al. 1999b). Pexiganan was synthesized through a series of substitutions and deletions of amino acid residues and it displayed a broad-spectrum antimicrobial activity against both Gram-positive and Gram-negative bacteria. The MSI-78 was primarily developed as a therapeutic agent for the treatment of infected diabetic foot ulcers and the Phase III of clinical trials were already finished in October 2016.

To date, many methods have been developed to design peptides with improved biological activity, such as: substitutions and deletions of amino acids, determination of structure–activity relationships, e.g., altering secondary structure and stabilization of α-helix (Fosgerau and Hoffmann 2015). Another approach to improve the peptide antimicrobial properties is reversion of the sequence. In other words, a retro analog has the same configuration of chiral centers but an opposite rank order of amino acid residues. However, this procedure not always results in enhanced antimicrobial activity. Conversely, it may cause a decrease in selectivity and increase in toxicity (Subbalakshmi et al. 2001). The increased antimicrobial activity of retro analogs has been reported in the literature. For example, Subbalakshmi et al. (2001) investigated biological activity of synthetic analogs of SPFK peptide (PKLLKTFLSKWIG), which creates the most hydrophobic fragment of bovine seminal plasmin. In this study, biological activity of the retro- and diastereo-analogs with C-terminal acid or amide was investigated. It was found that the compounds with reversed sequence exhibited a comparable or even higher activity against the tested microorganisms, but their hemolytic activity was marginal. Another study conducted by Gopal et al. (2011) focused on the activity of an analog of (WK)_3_ peptide. The peptide with the reversed sequence exhibited twice as high activity against all tested microorganisms (Gram-positive, Gram-negative bacteria, and fungi) with a comparable non-hemolytic behavior. It should be emphasized that only a wide range of the tested strains may provide reliable assessment of peptide activity if potentially applied to a specific infection.

The aim of this study was to learn whether or not the retro analogs of the following peptides: aurein 1.2, CAMEL, citropin 1.1, omiganan, pexiganan and temporin A, exhibit different antimicrobial activity, hemolytic activity, hydrophobicity, secondary structure, and ability to self-association. The hydrophobicity was determined using the RP-HPLC system. Retention of peptides is directly related to their hydrophobicity and hence retention time (or organic phase content) is a parameter that characterizes this feature. Moreover, to assess peptide self-association, an RP-HPLC-temperature profile was determined. It is believed that peptide self-association facilitates retention in RP-HPLC; however, both monomeric and self-associated species occur simultaneously. When a self-associated form occurs, an increase in temperature results in its disruption and consequently longer retention time is observed. Peptide tendency to self-association may be characterized by a maximum (*T*_max_) on a normalized plot of the change in retention time at different temperatures. Obviously, increasing temperature causes a decrease in mobile phase viscosity and increase in mass transfer between the mobile and stationary phase. These contribute to accelerated retention and stand in contrast to the effect caused by disruption of self-associates. At *T*_max_ the effect of self-associate disruption on retention time is equal to effect of increase in mass transfer and lowered viscosity (Lee et al. 2003).

## Materials and methods

### Peptide synthesis

The peptides (names and sequences in Table 1) were synthesized manually by solid-phase method using Fmoc chemistry on polystyrene resin modified by a Rink amide linker (Fields and Noble 1990). Single deprotection of the Fmoc group was carried out in a 20% (v/v) piperidine (Merck, Darmstadt, Germany) solution in *N,N*-dimethylformamide (DMF; Honeywell, Seelze, Germany) for 15 min. Acylation with protected amino acid was conducted in a DMF/DCM solution (DCM-dichloromethane; Chempur, Piekary Slaskie, Poland) with coupling agents for 1.5 h using a threefold molar excess of *N,N′*-diisopropylcarbodiimide (DIC; Peptideweb, Zblewo, Poland) and OxymaPure (Iris Biotech GmbH, Marktredwitz, Germany). Every step was preceded by rinsing the resin and running the chloranil test. Peptides were cleaved from the resin using one of the mixtures; (A)—trifluoroacetic acid (TFA; Apollo Scientific, Denton, UK), phenol, triisopropylsilane (TIS; Sigma-Aldrich, St. Louise, MO, USA), and water (92.5:2.5:2.5:2.5 v/v/v/v); (B)—trifluoroacetic acid, triisopropylsilane, and water (95:2.5:2.5 v/v/v). Mixture (A) was used with peptides containing a tryptophan residue, whereas mixture (B) for the remaining peptides. Crude peptides were precipitated with cold diethyl ether (Chempur, Piekary Slaskie, Poland) and lyophilized. Subsequently, the peptides were purified by reversed-phase high-performance liquid chromatography (RP-HPLC) with LP-chrom software. Purifications were carried out on a Phenomenex Gemini-NX C18 column (21.20 × 100 mm, 5.0 µm particle size, 110 Å pore size). UV detection at 214 nm was used, and crude peptides were eluted with a linear 10–70% acetonitrile gradient in deionized water over 90 min at room temperature. The mobile phase flow rate was 10.0 mL/min. Both eluents contained 0.1% (v/v) of TFA. Fractions were analyzed on a Waters X-Bridge Shield RP-18 column (4.6 × 150 mm, 3.5 µm particle size, 130 Å pore size) with UV detection at 214 nm. Pure fractions (>95%, by HPLC analysis) were collected and lyophilized. The identity of all compounds was confirmed by mass spectrometry (ESI–MS).

**Table 1:** Peptides used in this study

Peptide	Sequence	Average mass (Da)	Net charge	MS analysis		
				*z* ^a^	*m*/*z* ^b^	*m*/*z* ^c^
aurein 1.2	GLFDIIKKIAESF-NH_2_	1479.78	+1	2	740.9	740.7
				3	494.3	494.3
r-aurein 1.2	FSEAIKKIIDFLG-NH_2_	1479.78	+1	2	740.9	740.9
				3	494.3	494.4
CAMEL	KWKLFKKIGAVLKVL-NH_2_	1770.33	+6	2	886.2	886.1
				3	591.1	591.3
				4	443.6	443.9
r-CAMEL	LVKLVAGIKKFLKWK-NH_2_	1770.33	+6	2	886.2	886.0
				3	591.1	591.0
				4	443.3	443.6
				5	355.1	355.1
citropin 1.1	GLFDVIKKVASVIGGL-NH_2_	1614.99	+2	2	808.5	808.5
				3	539.3	539.5
r-citropin 1.1	LGGIVSAVKKIVDFLG-NH_2_	1614.99	+2	2	808.5	808.4
				3	539.3	539.4
omiganan	ILRWPWWPWRRK-NH_2_	1779.17	+5	2	890.6	890.3
				3	594.1	594.0
				4	445.8	445.8
r-omiganan	KRRWPWWPWRLI-NH_2_	1779.17	+5	2	890.6	890.5
				3	594.1	594.1
				4	445.8	445.9
pexiganan	GIGKFLKKAKKFGKAFVKILKK-NH_2_	2477.21	+10	2	1239.6	1239.8
				3	826.7	826.6
				4	620.3	620.4
				5	496.5	496.6
				6	413.9	414.0
r-pexiganan	KKLIKVFAKGFKKAKKLFKGIG-NH_2_	2477.21	+10	3	826.2	826.6
				4	619.9	620.4
				5	496.1	496.5
				6	413.6	414.0
temporin A	FLPLIGRVLSGIL-NH_2_	1396.78	+2	2	699.4	699.3
r-temporin A	LIGSLVRGILPLF-NH_2_	1396.78	+2	2	699.4	699.5

^a^ Ion charge

^b ^Calculated mass to charge ratio

^c ^Measured mass to charge ratio

### Ion chromatography

Determination of trifluoroacetate (TFA^−^) content was performed by ion chromatography (Dionex ICS-5000+, Thermo Scientific). Isocratic elution was performed with 4.5 mM Na_2_CO_3_ and 1.4 mM NaHCO_3_ in water and a flow rate of 1.2 mL/min. Detection was done by suppressed conductivity with ASRS 300—Anion Self-regenerating suppressor and suppressor current of 31 mA. Column characteristics—Dionex IonPac AS22, dimensions 4 × 250 mm, column compartment temperature 30 °C and detector temperature 35 °C. The samples were dissolved in water to obtain a concentration of 0.5 mg/mL and injection volume was 20 µL. The validation of the method was performed in accordance with the ICH guidelines Q2(R1) (ICH 2005).

### Organisms and antimicrobial assay

The minimal inhibitory concentrations (MICs) for bacteria and fungi were determined by the broth microdilution method according to the Clinical and Laboratory Standards Institute (CLSI) protocol (Clinical and Laboratory Standards Institute (CLSI) 2002, 2012). For this purpose, the initial inoculums of bacteria (5 × 10^5^ CFU/mL) in Mueller–Hinton Broth (Biocorp, Warsaw, Poland) were exposed to the ranging concentrations of compounds (0.125–256 µg/mL) and incubated for 18 h at 37 °C. In case of fungi, the initial inoculums of 2 × 10^3^ CFU/mL in RPMI-1640 (Sigma-Aldrich, Steinheim, Germany) were exposed to the ranging concentrations of compounds (0.5–256 µg/mL) and incubated for 24 h at 37 °C. The experiments were conducted on 96-well microtiter plates, with the final volume of 100 µL. Cell densities were adjusted spectrophotometrically (Multiskan™ GO Microplate Spectrophotometer, Thermo Scientific) at the wavelengths of 600 nm for bacteria and 530 nm for fungi. The MIC was taken as the lowest drug concentration at which a noticeable growth of microorganisms was inhibited. Reference strains of bacteria: *Escherichia coli* ATCC 25922, *E. coli* ATCC 23506, *Enterococcus faecalis* PCM 2673, *Klebsiella pneumoniae* ATCC 700603, *Pseudomonas aeruginosa* ATCC 9027, *Staphylococcus aureus* ATCC 25923, *S. aureus* 9144, *Streptococcus pneumoniae* ATCC 49619, and fungi: *Aspergillus niger* ATCC 16404, *Candida albicans* ATCC 10231, and *C. glabrata* ATCC 15126 were obtained from Polish Collection of Microorganisms (PCM, Polish Academy of Sciences, Wroclaw, Poland) and from American Type Culture Collection (ATCC). All experiments were conducted in triplicate.

### Hemolysis assay

The hemolysis assay was conducted according to the procedure described previously in the literature (Avrahami and Shai 2004). Fresh human red blood cells (RBCs) with EDTA as anticoagulant were rinsed three times with a phosphate-buffer saline (PBS) by centrifugation at 800×*g* for 10 min and resuspended in PBS. Serial dilution of peptides (1–512 µg/mL) was conducted in PBS on 96-well plates. Then the stock solution of RBCs was added to reach a final volume of 100 µL with a 4% concentration of erythrocytes (v/v). The control wells for 0 and 100% hemolysis consisted of RBCs suspended in PBS and 1% of Triton-X 100, respectively. Subsequently, the plates were incubated for 60 min at 37 °C and then centrifuged at 800×*g* for 10 min at 4 °C (Sorvall ST 16R Centrifuge, Thermo Scientific). After centrifugation the supernatant was carefully resuspended to new microtiter plates and the release of hemoglobin was monitored by measurement of absorbance at 540 nm (Multiskan™ GO Microplate Spectrophotometer, Thermo Scientific). All experiments were conducted in triplicate.

### CD experiments

CD spectra of the peptides in the buffered (10 mM phosphate buffer, pH 7.4) aqueous micellar solutions of SDS (sodium dodecyl sulfate) and DPC (dodecylphosphocholine) were acquired using a Jasco J-815 spectropolarimeter. All measurements were conducted using 0.15 mg/mL peptide solutions at 298 K. Experiments were performed in triplicate to increase signal-to-noise ratios and carried out over the 195–260 nm range. Final spectra were corrected by background subtraction and analyzed as a mean residue molar ellipticity, MRME (degree × cm^2^ × dmol^−1^) *vs* wavelength *λ* (nm). The helicity fraction (fH) was calculated according to the Eq. (1) (Rao et al. 2013).

1 $$fH = \frac{{\theta_{222} - \theta_{\text{c}} }}{{\theta_{222} \infty - \theta_{\text{c}} }},$$

where *θ*_c_ = 2220 − (53 × *T*) and *θ*_222∞_ = (−44,000 + 250 × *T*) × (1 − *k*/Nr), *k* is the wavelength-dependent constant (at 222 nm, *k* = 2.4), Nr is the number of residues, and *T* is the temperature expressed in °C.

### Determination of peptide hydrophobicity parameters

To determine peptide hydrophobicity, the RP-HPLC system was used. The equipment used was Waters Alliance e2695 system with a Waters 2998 PDA Detector (software-Empower^®^3). All analyses were carried out on a Waters X-Bridge Shield RP-18 column (4.6 × 150 mm, 3.5 µm particle size, 130 Å pore size) and each peptide sample was analyzed in triplicate. UV detection at 214 nm was used, and samples (10 µL) were eluted with a linear 20–50% acetonitrile gradient in deionized water over 30 min at 25.0 ± 0.1 °C (gradient 1% of ACN/min). The mobile phase flow rate was 0.5 mL/min. Both eluents contained 0.1% (v/v) of TFA. The peptides were dissolved in water (0.1% TFA, v/v) to obtain a concentration of 1 mg/mL. The hydrophobicity was expressed as acetonitrile content in the mobile phase at a retention time of a peptide (*t*_R_). The dead time (*t*_0_) was determined by injecting water and measuring the elution time of negative peaks. To calculate hydrophobicity, Eq. (2) was used.

2 $$Y_{{\% {\text{ACN}}}} = \frac{{1\% {\text{ACN}}}}{\hbox{min} }(t_{\text{R}} - t_{0} ) + 20\% {\text{ACN}},$$

where *Y* _%ACN_ is the acetonitrile content [%] at the peak maximum; *t*_R_ is the retention time [min]; *t*_0_ is the dead time [min]; 1% ACN/min is the organic phase gradient; 20% ACN is the content of organic solvent at the beginning of method.

### Temperature profiling in reversed-phase chromatography: measurement of self-association

To determine retention time of the peptides in a temperature gradient, RP-HPLC system was used (Lee et al. 2003). For this purpose, a Waters Alliance e2695 separation module with thermostat was applied. Mobile phase was acetonitrile and water, both containing 0.1% TFA. Column characteristics: Waters X-Bridge C18, dimensions 3 × 100 mm, particle size 3.5 µm, 130 Å pore size. The method used in all analyses was 10–60% of ACN in 40 min at a flow rate of 0.5 mL/min and absorption measurement at 214 nm. Temperature range was 5–80 °C with 5 °C steps with the accuracy of ±0.1 °C. Retention time was determined at each temperature. Peptide concentration was 1 mg/mL and injection volume was 10 µL. All analyses were conducted in triplicate.

## Results and discussion

### Ion chromatography: counter-ion content

After synthesis and purification steps of SPPS the peptides were obtained as trifluoroacetate salts. TFA^−^ can be found in peptides lyophilizates in two forms: as counter-ions to a positively charged side groups of peptides and as a free trifluoroacete. TFA can affect conformation of peptides via pH and consequently their biological activity (Cinelli et al. 2001; Wada et al. 2003). TFA is also known for its toxicity, e.g., to eukaryotic cells (Cornish et al. 1999; Pini et al. 2012). We determined the content of TFA^−^ in peptides and their retro analogs by ion chromatography using the validated procedure. All measurements were made in triplicate for two separate samples. For all pairs of the peptide and its retro analog the difference in the TFA^−^ level was below 10%. The results show that there is an insignificant difference between the peptides and their retro analogs that could influence conformation and biological activity.

### Antimicrobial assay and hemolytic activity

**Fig. 1:**
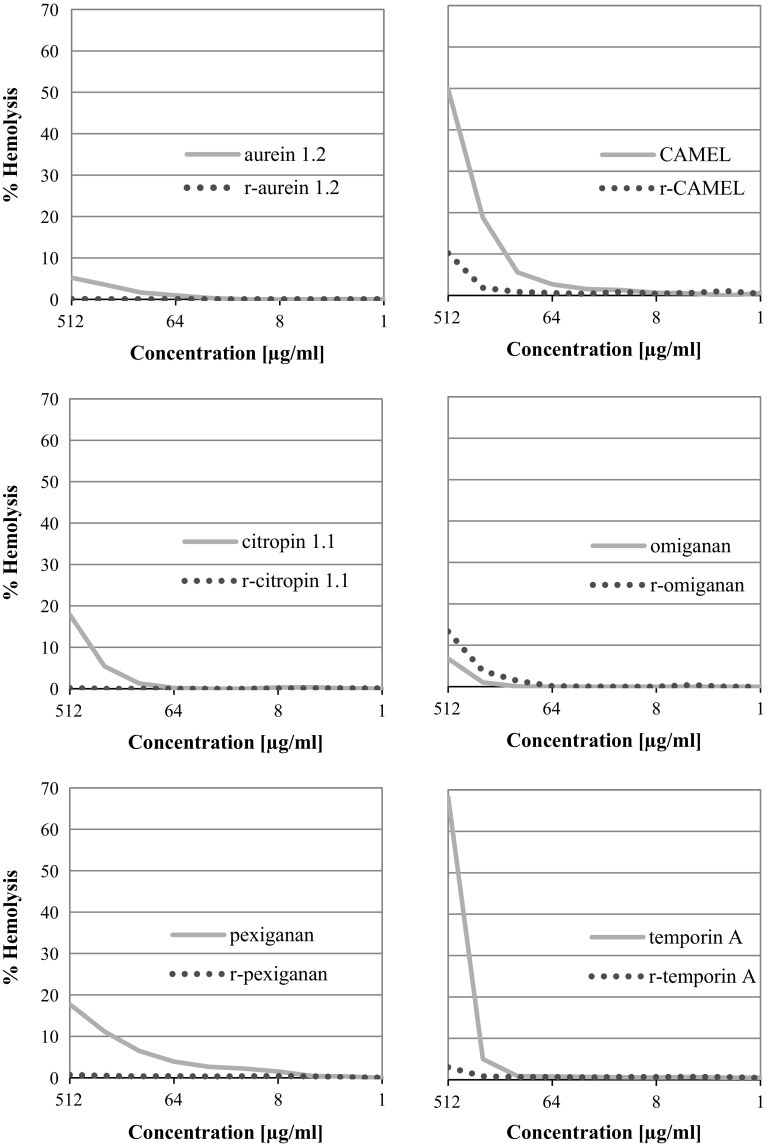
Percentage of hemolysis of erythrocytes vs peptide concentration

In the present study, the majority of compounds exhibited antimicrobial activity (Table 2). However, it was found that only two analogs revealed enhanced activity, as compared to that of the parent compounds. The results supported the concept that the synthesis of retro analogs might be the source of novel AMPs with promising properties. Importantly, the reduced hemolytic activity correlates with lower antimicrobial activity. To maintain the consistence of the discussion, both antimicrobial and hemolytic activities (Fig. 1) will be discussed in pairs.

**Table 2:** MIC values (µg/mL) of the peptides and their retro analogs against reference strains of microorganisms

	aurein 1.2	r-aurein 1.2	CAMEL	r-CAMEL	citropin 1.1	r-citropin 1.1	omiganan	r-omiganan	pexiganan	r-pexiganan	temporin A	r-temporin A
Gram-positive bacteria												
*E. faecalis* PCM 2673	64	256	8	64	32	128	16	16	16	64	64	256
*S. aureus* ATCC 25923	128	>256	4	128	16	64	16	8	8	128	4	64
*S. aureus* ATCC 9144	128	>256	4	64	16	64	16	8	8	64	8	64
*S. pneumoniae* ATCC 49619	64	256	0.5	128	32	128	8	8	1	4	>256	>256
Gram-negative bacteria												
*E. coli* ATCC 25922	128	256	2	128	32	64	16	8	4	8	256	256
*E. coli* ATCC 23506	128	256	1	64	32	128	32	4	2	2	>256	256
*K. pneumoniae* ATCC 700603	16	128	0.125	2	16	32	8	4	1	0.25	128	128
*P. aeruginosa* ATCC 9027	256	>256	2	8	128	>256	16	4	2	2	>256	256
Fungi												
*A. niger* ATCC 16404	64	>256	16	256	64	256	64	64	16	32	256	256
*C. albicans* ATCC 10231	128	>256	32	256	128	128	128	32	256	256	256	256
*C. glabrata* ATCC 15126	128	>256	64	>256	128	256	128	64	256	128	>256	>256

#### Aurein 1.2 and r-aurein 1.2

Aurein 1.2 was one of the least effective compounds in this study in terms of minimal inhibitory concentrations. The growth of the majority of microorganisms was inhibited at 128 µg/mL. The highest activity was found against *K. pneumoniae* (16 µg/mL), while the lowest was against *P. aeruginosa* (256 µg/mL). Similar results were obtained in previous works conducted by our research group concerning the activity of amphibian AMPs (Kamysz and Nadolski 2005). In another research performed on clinical strains of *S. aureus,* the MIC ranged between 32 and 128 µg/mL (Baranska-Rybak et al. 2011). However, studies on a broader range of cocci isolated from nosocomial infections carried out by Giacometti et al. (2007) indicated a lower activity of aurein 1.2 with a high bactericidal effect in a time-killing assay. Another parameter measured in this study was the rate of hemolysis which was lower than 10% at MIC concentrations. As a matter of fact, the antimicrobial activity of the retro analog was the least in that study. R-aurein 1.2 did not inhibit the growth of fungi. Moreover, the highest activity was observed against *K. pneumoniae* (128 µg/mL). Additionally, the hemolysis of erythrocytes in the presence of r-aurein 1.2 was one of the lowest.

#### CAMEL and r-CAMEL

In contrast to aurein 1.2, CAMEL was found to be the strongest antimicrobial in this study. It was very active against all groups of microorganisms: Gram-positive, Gram-negative bacteria, and fungi. For the first group the highest activity was found against *S.* *pneumoniae*, whereas for the second, it was highly active against *K.* *pneumoniae* (0.125 µg/mL). The growth of fungi: *A. niger*, *C.* *albicans* and *C. glabrata*, was inhibited at 16, 32 and 64 µg/mL, respectively. The high antimicrobial activity was not maintained in case of retro analog. r-CAMEL was significantly less active against the majority of microorganisms than the parent molecule—up to 256 times less active against *S.* *pneumoniae*, 16-fold less active against *K. pneumoniae,* and 4-fold less against *P. aeruginosa*. Despite this fact, the hemolysis rate of erythrocytes was below 10% at MIC concentrations. Interestingly, Merrifield et al. (1995) also conducted the study on antimicrobial activity of retro- and retro-enantio analogs of cecropin-melittin fragments. However, the antibacterial activity was determined by inhibition-zone assay on agarose plates, thus providing rather qualitative than quantitative results. This notwithstanding, the retro analog exhibited a suppressed activity. Our quantitative study, conducted by broth microdilution method has shown that the activity of r-CAMEL against some organisms is still high, being, however, at least 4 times lower than that of the parent peptide.

#### Citropin 1.1 and r-citropin 1.1

Citropin 1.1 is another antimicrobial peptide derived from amphibians. In our study, this peptide was characterized by a relative good activity against Gram-positive and Gram-negative bacteria, except *P.* *aeruginosa* (16–32 µg/mL). However, it exhibited a weak (*A. niger*) or virtually no antifungal activity (*Candida* species). Similar activity of citropin 1.1 was found in previous research focused on physical and biological examination of truncated fragments of the peptide (Sikorska et al. 2009). The retro analog was fourfold less active than the parent molecule with no activity against *P.* *aeruginosa*. In contrast to citropin 1.1, the hemolysis in case of r-citropin 1.1 was almost unnoticed.

#### Omiganan and r-omiganan

Omiganan is an example where the sequence reversion caused a significant improvement in antimicrobial activity against Gram-negative bacteria and fungi (except *A. niger*). With Gram-positive strains, the MIC values ranged between 8 and 16 µg/mL. The distinct increase in the activity was observed in case of *E. coli* ATCC 23506 and *P. aeruginosa*. For these strains, the inhibitory concentrations were 4-times lower than that of the parent compound. Similar results for omiganan were reported by Sader et al. (2004). In their study, the omiganan pentachloride was examined and more than 1500 clinical isolates of bacteria and fungi were assessed in terms of antimicrobial activity. The hemolytic activity of r-omiganan was also distinctive due to its higher toxicity in comparison to that of the parent molecule; while in other pairs the change in hemolytic activity was different. In other words retro analogs were less hemolytic.

#### Pexiganan and r-pexiganan

Pexiganan was the second compound with relatively high antimicrobial activity, but with no potency against *Candida* strains (256 µg/mL). A similar antimicrobial efficacy against various pathogens was previously reported by other research groups (Lopez-Medina et al. 2015). Moreover, other studies focused on antimicrobial potency against strains isolated from diabetic foot ulcers have also proved similar activities against a wide spectrum of pathogens (Ge et al. 1999a; Flamm et al. 2015). However, our study indicated high potency against *S. pneumoniae*, *E. coli*, *K. pneumoniae*, and *P. aeruginosa* strains (1–4 µg/mL). In contrast to other retro analogs, r-pexiganan exhibited an enhanced activity only against Gram-negative strain *K. pneumoniae*. Moreover, r-pexiganan did not cause a noticeable hemolysis. For this reason, it can be hypothesized that r-pexiganan might offer a great potential for the treatment of infections caused by Gram-negative bacteria.

#### Temporin A and r-temporin A

Temporin A did not reveal any significant activity against Gram-negative bacteria and fungi. However, it was potent against *S.* *aureus* strains (4–8 µg/mL). A decreased activity of r-temporin A was also observed, but against some strains it remained at the same unsatisfactory level. The results of our previous work on the antimicrobial activity of retro analog of temporin A are consistent with present study findings (Kamysz et al. 2006). Also the rate of hemolysis caused by temporin A was the highest among all of the tested compounds.

### CD results

**Fig. 2:**
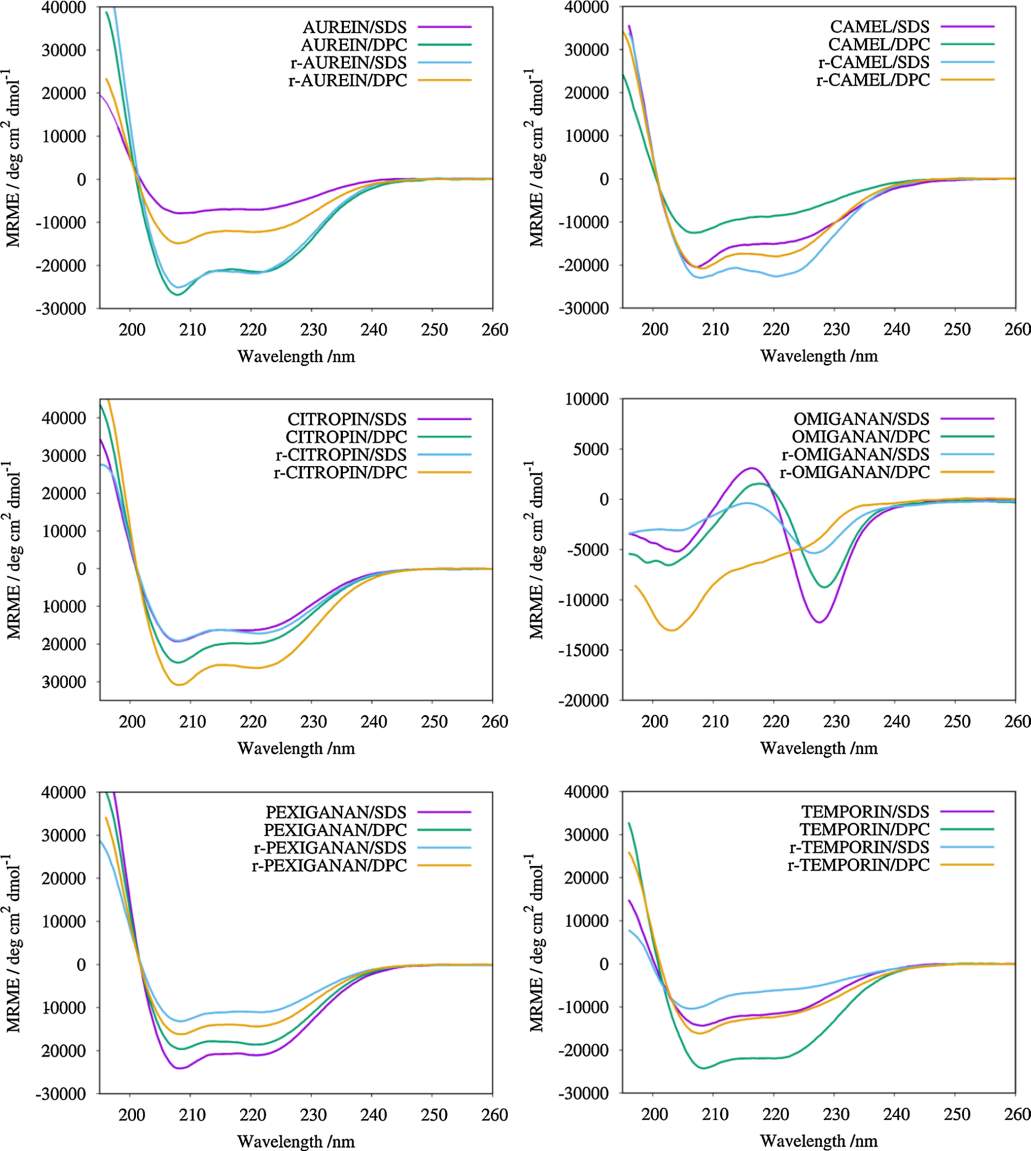
Far-UV CD spectra of parent peptides and their retro counterparts in SDS and DPC micellar solutions. *MRME* mean residue molar ellipticity

The CD spectra of the peptides were recorded in two micellar environments, the SDS and DPC, the popular membrane models of anionic and zwitterionic membranes, respectively. It is known that CD negative bands at 222 and 208 nm are indicative of α-helical structure with the 222/208 ratio describing the likelihood that the helix is formed either in isolation or within a coiled-coil structure. The ratio higher than 1 usually indicates a coiled-coil structure formed, while that less than 0.9 indicates the presence of isolated helices. As seen in Fig. 2, all the peptides, except omiganan and r-omiganan, adopt more or less α-helical conformation in both membrane-like micellar solutions. The helicity fractions fall in a wide range of 22–82% (Table 3). With pexiganan and temporin A, both the antimicrobial activity against Gram-positive strains and α-helical structure content decreases upon reversing amino acid sequence. In turn, with aurein 1.2, citropin 1.1, and CAMEL, there is suppressed antimicrobial activity and an increase in helical propensity (in SDS solution).

**Table 3:** *θ*_222_/*θ*_208_ ratio and helicity fraction (*f*_H_) of the peptides in DPC and SDS

Peptides	SDS		DPC	
	*θ*_222_/*θ*_208_	Helicity fraction (*f*_H_)	*θ*_222_/*θ*_208_	Helicity fraction (*f*_H_)
aurein 1.2	0.89	25	0.80	71
r-aurein 1.2	0.86	71	0.82	41
CAMEL	0.73	48	0.67	28
r-CAMEL	0.97	71	0.85	57
citropin 1.1	0.83	51	0.79	62
r-citropin 1.1	0.90	55	0.85	82
pexiganan	0.87	63	0.95	56
r-pexiganan	0.84	35	0.89	44
temporin A	0.79	38	0.90	71
r-temporin A	0.60	22	0.74	41

Among the peptides studied, r-omiganan revealed enhanced antimicrobial activity as well as stronger hemolytic properties as compared to that of the parent molecule. The CD spectra of omiganan in both the SDS and DPC solutions show a shallow minimum around 200 nm and a deeper one at 230 nm. The former is characteristic of an unordered structure, whereas the latter results from interactions between the side-chains of tryptophan, as suggested by Faccone et al. (2014). A similar CD spectrum, but with distinctly lower intensity, was recorded for r-omiganan in SDS micellar solution. In turn, the CD spectrum of r-omiganan was significantly modified in the presence of DPC, where the 200 nm band was strengthened, while that at 230 nm was attenuated. These differences are induced either by changes in electrostatic and hydrophobic peptide–micelle interactions or/and interactions between Trp chromophores upon reversing the sequence.

**Fig. 3:**
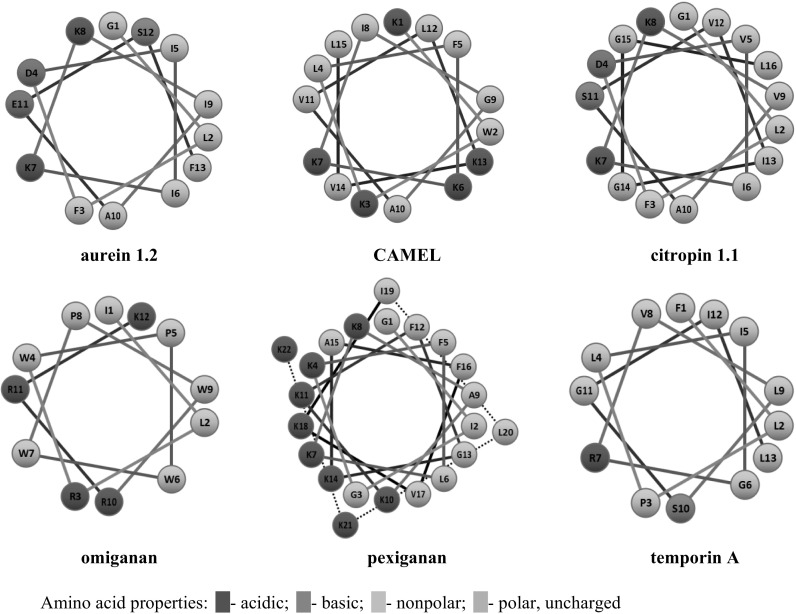
Helical-wheel projection of the tested peptides

Omiganan does not display a helical structure, and for this reason a comparison of MRME at 208 and 222 nm is irrelevant. According to the *θ*_222_/*θ*_208_ ratio it is concluded that none of the tested peptides exhibit the coiled-coil structure. However, the highest values refer to r-CAMEL in SDS environment (0.97) and to pexiganan in DPC medium (0.94). Porcelli et al. (2006) reported pexiganan to form a partially distorted α-helix in a short coiled-coil structure. The calculated fH values indicate that the retro compounds of CAMEL and citropin 1.1 adopt more helical structure in both environments. Furthermore, pexiganan, temporin A, and their analogs exhibited opposite relation. Helical structure of the aurein 1.2, citropin 1.1, and temporin A, was confirmed also in other studies (Rozek et al. 2000a, b; Kamysz et al. 2006). Helical-wheel projections of the tested peptides are presented in Fig. 3. This kind of projection enables to detect the occurrence of hydrophobic and hydrophilic faces of a α-helical peptide.

### Determination of peptide hydrophobicity parameters

The results clearly identify significant differences in retention time among the compounds studied. In RP-HPLC, the retention of molecules depends on their hydrophobicity. In effect, hydrophobic molecules elute later than do hydrophilic ones. The hydrophobicity is expressed as a percentage of ACN seemingly being more universal than retention time. A comparison between peptides and their retro analogs indicates that the sequence of amino acids (their rank order) is critical for interaction with stationary phase (RP-HPLC), even if the composition of the molecules is identical (Table 4). In reference to other pairs (peptide vs retro analog), omiganan exhibited a different pattern with a retro compound being more hydrophobic. Omiganan exhibited higher hydrophilicity and simultaneously lower antimicrobial activity, except r-omiganan. Interactions between peptides and lipid membrane core of pathogens were found to be crucial for their antimicrobial activity. Bearing in mind that the core of lipid membrane is hydrophobic, it seems reasonable to claim that increased hydrophobicity should have an influence on this interaction. Variations in hydrophobicity significantly correlate with antimicrobial activity but only if investigated pairs are considered. In fact, the results do not confirm any convincing relation for all the tested compounds as a general rule in this area, but indicate that the increased hydrophobicity in retro analogs strengthen their antimicrobial activity. Kim et al. (2005) reported a correlation between the α-helical antimicrobial peptides retention time in RP-HPLC (hydrophobicity) and their antimicrobial activity against *S.* *aureus*, while there was no such tendency in the case of the Gram-negative strain, *E. coli.*

**Table 4:** Hydrophobicity parameters (RP-HPLC) of the different peptides

Peptide	*t*_R_ (min)	% ACN	Δ %ACN^a^
aurein 1.2	23.93	43.93	6.83
r-aurein 1.2	17.11	37.10	
CAMEL	13.73	33.73	3.02
r-CAMEL	10.71	30.71	
citropin 1.1	22.40	42.40	1.12
r-citropin 1.1	21.28	41.28	
omiganan	12.92	32.92	−2.56
r-omiganan	15.48	35.48	
pexiganan	10.58	30.58	4.22
r-pexiganan	6.36	26.36	
temporin A	22.80	42.80	3.89
r-temporin A	18.91	38.91	

^a^Δ %ACN = %ACN_peptide_ − %ACN_retro analog_; the standard deviation of *t*_R_ did not exceed 0.05

### Temperature profiling in reversed-phase chromatography

**Fig. 4:**
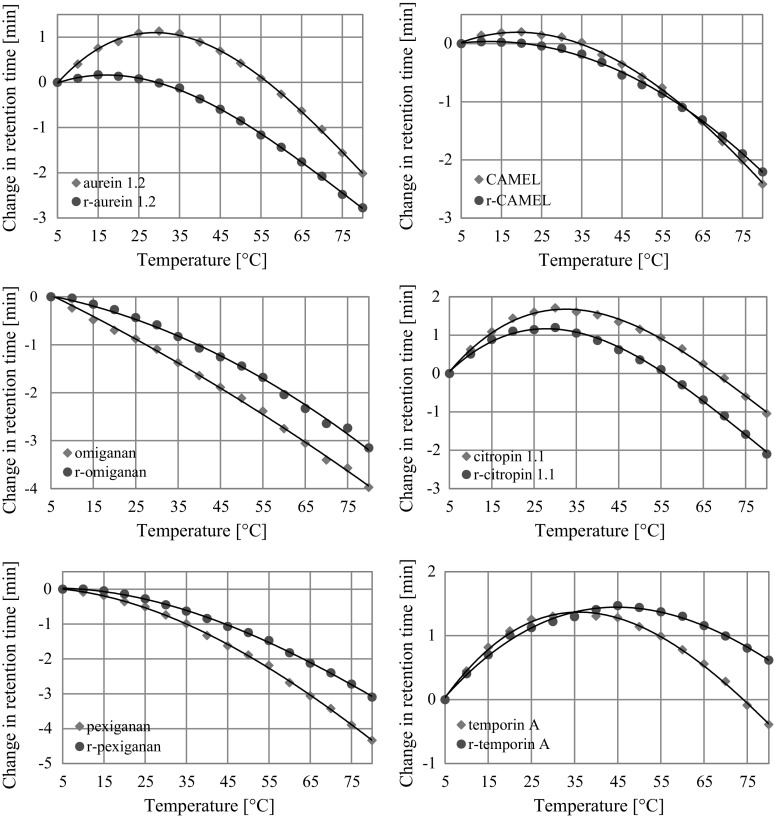
Normalized RP-HPLC—temperature profiles of different peptides (*blue squares*) and their retro analogs (*red circles*)

The temperature profiles of the different peptide and retro analog pairs are presented in the Fig. 4 as a change in retention time over temperature referred to 5 °C. Normalization can be expressed as Change in retention time = *t*_R(*X* °C)_ − *t*_R(5 °C)_.

#### Aurein 1.2 and r-aurein 1.2

It has been documented that aurein 1.2 possesses α-helical structure and a well-defined hydrophilic and hydrophobic surface (Rozek et al. 2000a, b). The proposed mode of action of aurein 1.2 is based on a carpet-like mechanism and peptide aggregation improves its antimicrobial activity (Shahmiri et al. 2015). Assuming that peptide–peptide interaction plays a pivotal role in membrane disruption, it should be noticed that aurein 1.2 exhibited both higher antimicrobial activity and augmented tendency to self-association than did its retro analog due to its higher *T*_max_ value (temperature at which maximum retention in RP-HPLC temperature profiling is observed, Table 5).

**Table 5:** *T*_max_ values

Peptide	*T*_max_ (°C)
aurein 1.2	30
r-aurein 1.2	15
CAMEL	20
r-CAMEL	10
citropin 1.1	30
r-citropin 1.1	30
temporin A	35
r-temporin A	45

#### CAMEL and r-CAMEL

The normalized temperature profile for CAMEL and its retro analog shows two different maxima (Fig. 4; Table 5). The higher temperature at which the maximum emerges and the more distinct change in retention time of CAMEL indicate enhanced tendency to form self-associates. Moreover, CD results revealed that both CAMEL and r-CAMEL adopt α-helical structure in DPC and SDS environment. However, other studies reported a monomeric state when the molar lipid/peptide ratio was high (>120/1) (Bhargava and Feix 2004). This finding does not contradict to propose self-association at higher concentrations. After linking together, the difference in *T*_max_ value with antimicrobial activity of CAMEL, it should be stated that improved interaction between peptide molecules may have been observed as a higher antimicrobial activity. Simultaneously, it cannot be underestimated that CAMEL in applied conditions shows higher hydrophobicity than its retro analog.

#### Citropin 1.1 and r-citropin 1.1

The temperature profiles of citropin 1.1 and its retro analog are comparable. They show a maximal retention time at 30 °C. This result indicates that the analyzed pair of peptides forms self-associates of similar characteristics. Self-association study, in contrast to hydrophobicity analysis, does not reveal any significant difference between the peptides. In this case, the reversion of amino acid sequence has a slight or no impact on the tendency to self-association.

#### Omiganan and r-omiganan

Omiganan shows a linear correlation instead of a slightly curved line as does r-omiganan. This fact indicates that omiganan molecules do not significantly interact with each other and do not represent a strictly defined amphiphilic structure. This statement found confirmation in CD results, where the peptide pair could not be identified as α-helix. Other studies also provide evidence that omiganan does not adopt helical structure (Faccone et al. 2014). Actually, omiganans primary structure (ILRWPWWPWRRK-NH_2_) is similar to that of indolicidins (ILPWKWPWWPWRR-NH_2_), which has an unordered structure containing a poly-l-proline-type II helix and β-turn. It is highly probable that both expose a similar wedge-shaped secondary structure (in DPC) (Rozek et al. 2000a, b). Regarding the temperature profile, we can only hypothesize that r-omiganan is likely to occur as a self-associate and hence its interaction with bacterial membrane would be different in comparison to that of its parent molecule. Self-association of the molecules may arise from interaction between hydrophobic surfaces of the peptide. Usually, antimicrobial α-helical peptides have hydrophilic amino acid residues on the opposite face of the helix in relation to the hydrophobic amino acid residues. Surprisingly, indolicidin and its retro analog were shown to have similar structural properties and antimicrobial activity against *Salmonella typhimurium* (Nagpal et al. 2002). However, stronger activity against *Bacillus subtilis* and *E. coli* and a lower hemolysis of r-indolicidin were also reported (Ando et al. 2010).

#### Pexiganan and r-pexiganan

Pexiganan and its retro analog do not exhibit significant maxima on the normalized temperature profile. The CD spectra indicate that the peptide adopts α-helical structure in both the SDS and DPC solutions. Considering this and the helical-wheel projection of pexiganan (Fig. 3), it is reasonable to expect interaction between hydrophobic faces of the peptide. Presumably, this interaction manifests as a curved line on the profile. In fact, secondary structure of pexiganan is solvent-dependent (Shanmugam et al. 2005), and in the hydrophilic environment it appears as a random coil with β-turn. In organic solvents, such as trifluoroethanol, this peptide adopts a α-helix conformation. Additionally, pexiganan has been reported to form antiparallel dimer in the presence of DPC and the “phenylalanine zipper” plays a key role in this interaction (Porcelli et al. 2006). One might claim that RP-HPLC conditions were insufficient to provide effective self-association due to mobile phase gradient, which resulted in unstructured state of pexiganan. However, RP-HPLC conditions should induce peptide secondary structure (Blondelle et al. 1995). Somehow, the results suggest those interactions might have taken place but do not differentiate them between the peptides.

#### Temporin A and r-temporin A

Undoubtedly, temporin A adopts a helical structure in hydrophobic environment of TFE as was confirmed previously (Mangoni et al. 2000). However, r-temporin A adopts less helical structure. This is consistent with previous findings (Kamysz et al. 2006, 2007). The results obtained for temporin A and its retro analog show that a defined secondary structure is not *conditio sine qua non* for triggering significant interactions between the peptide molecules. Even the reduced helicity does not hamper those interactions. Considering the helical-wheel projection of temporin A (Fig. 3) and its amino acid composition, the conclusion seems to be more convincing. Since temporin A consists mainly of hydrophobic amino acids, it does not require any particular structure to promote hydrophobic interactions. In other words, the positions of hydrophilic serine and basic arginine residues, resulting from the peptides’ secondary structure, seem to be irrelevant and does not have a disruptive character. Furthermore, Saravanan et al. (2013) suggest that temporin A forms head-to-tail dimers and higher oligomeric state structures in lipopolysaccharide micelles. Moreover, it has been found that dimers and aggregates of temporin A present in the lipopolysaccharide structure are in an antimicrobial inactive form understood as a type of bacterial protection. The *T*_max_ values for all tested peptides are presented below (Table 5).

Omiganan, pexiganan, and their retro analogs do not exhibit any maxima on the normalized RP-HPLC—temperature profile. Interestingly enough, helicity fraction (fH) in SDS (Table 3) and *T*_max_ value seem to be correlated. In the peptide pairs, ones with a higher fH simultaneously exhibited lower *T*_max_, except omiganan which does not adopt helical structure. Moreover, if fH are approximately equal the same is seen in the case of *T*_max_ value (citropin 1.1 vs r-citropin 1.1). However, no significant relationship was noted for fH determined in DPC. This may result from a closer similarity between SDS and physico-chemical character of the stationary phase. To clarify this statement, SDS has a negative charge and aliphatic chain (C12), whereas the solid phase contains partially protonated acidic silanol groups and aliphatic chains (C18). Again, DPC is zwitterionic. Concluding, despite the decreased helicity in SDS solution, the peptides exhibited an enhanced tendency to form self-associates.

All of the experiments helped to characterize peptides used in this study but did not explain the reason of the observed differences in hydrophobicity and antimicrobial activity between a peptide and its retro analog. Any structural change should be analyzed for better understanding of the source of the diversity. The synthesized peptides have the C-terminal amide and N-terminal amine groups. The amino and carboxyl functions of the amino acids are not equal, in terms of their basicity and acidity. Consequently, each terminal amino acid may have a slightly different impact on the peptide charge. It has been shown that hydrophobicity of the side-chain depends on amino acid position and chemical surroundings. Furthermore, *N* ^α^-amino group plays an essential role in the side-chain hydrophobicity of the N-terminal amino acid (Sereda et al. 1993). Importantly, each peptide bond has its own dipole moment. Owing to specific spatial orientation of dipoles, helix may also be characterized by its macrodipole which is a resultant of summation of dipoles of individual peptide bonds. Tripet et al. (2007) determined the influence of N- and C-terminus, and the location of amino acid residue in peptide on its side-chain hydrophobicity using RP-HPLC. The authors presented amino acid coefficients for prediction of peptide retention time. To assess the effect caused by amino acid residues changing their positions in retro analogs, appropriate values were adopted in this study to calculate overall impact on retention behavior. Presumably, the major effect resulted from modification of terminal amino acid residues. For example, peptides CDEF-NH_2_ and FEDC-NH_2_ are “retro-pair” and have opposite end-group amino acids; however, the difference between internal fragments seems to be less relevant (–CDEF– vs –FEDC–). The side-chain of D is surrounded by C and E in both cases, and the same with the side-chain of E which is permanently surrounded by D and F. Importantly, the reader should be aware that the presented concept is a considerable simplification for the purpose of calculations presented in Table 6. All calculations are based on the results obtained by Tripet et al. (2007). The influence of changing position (sequence reversion) on the retention time was assessed using N^α^-terminal amino and C^α^-terminal amide side-chain hydrophobicity coefficients at 25 °C. The effect of reversion was calculated as follows:

**Table 6:** Prediction of changes in retention time due to peptide sequence reversion

Parent peptide	N-terminal amino acid and effect of sequence reversion; Δ_1_ (min)		C-terminal amino acid and effect of sequence reversion; Δ_2_ (min)		Resulting change in retention; Δ_3_ (min)^a^
aurein 1.2	glycine	0.0	phenylalanine	−10.6	−10.6
CAMEL	lysine	−1.3	leucine	−10.2	−11.5
citropin 1.1	glycine	0.0	leucine	−10.2	−10.2
omiganan	isoleucine	+11.0	lysine	+1.3	+12.3
pexiganan	glycine	0.0	lysine	+1.3	+1.3
temporin A	phenylalanine	+10.6	leucine	−10.2	+0.4

^a^ Value should be interpreted as a general change, not as a particular one in retention time

$$\Delta_{1} = \left| {{\text{C}}^{\alpha } {\text{-terminal amide side}}-{\text{chain hydrophobicity coefficient}}} \right| - \left| {{\text{N}}^{\alpha } {\text{-terminal amino side}}-{\text{chain hydrophobicity coefficient}}} \right|$$

$$\Delta_{ 2} = \left| {{\text{N}}^{\alpha } {\text{-terminal amino side}} - {\text{chain hydrophobicity coefficient}}} \right| - \left| {{\text{C}}^{\alpha } {\text{-terminal amide side}} - {\text{chain hydrophobicity coefficient}}} \right|$$

$$\Delta_{3} = \, \Delta_{1} +_{{}} \Delta_{2}$$

For instance:

Aurein 1.2: GLFDIIKKIAESF-NH_2_.

Result of the sequence reversion (r-aurein 1.2): FSEAIKKIIDFLG-NH_2_

$$\Delta_{ 1} = \left| {{\text{C}}^{\alpha } {\text{-terminal amide side}}-{\text{chain hydrophobicity coefficient of glycine}}} \right| - \left| {{\text{N}}^{\alpha } {\text{-terminal amino side}}-{\text{chain hydrophobicity coefficient of glycine}}} \right|$$

$$\Delta_{ 2} = \left| {{\text{N}}^{\alpha } {\text{ - terminal amino side}} - {\text{chain hydrophobicity coefficient of phenylalanine}}} \right| - \left| {{\text{C}}^{\alpha } {\text{ - terminal amide side}} - {\text{chain hydrophobicity coefficient of phenylalanine}}} \right|$$

$$\begin{aligned} \Delta_{ 1} & = 0 \\ \Delta_{ 2} & = 2 2. 3{-} 3 2. 9= - 10. 6 {\text{ min}} \\ \Delta_{ 3} & = \Delta_{ 1} + \Delta_{ 2} = - 10. 6 {\text{ min}} .\\ \end{aligned}$$

The aim of the calculations was to learn whether the aspect of changes in side-chain hydrophobicity adequately explains the altering peptide retention behavior due to its sequence reversion. With aurein 1.2, citropin 1.1, CAMEL, and omiganan, the predictions are compatible with experimental results (Tables 4, 6). It should be emphasized that the calculated values, do not exactly describe the change in retention time, but rather represent a general relation between peptide and its retro analog. Nevertheless, pexiganan and temporin A exhibited a different relation with their retro analogs than that just predicted. This observation suggests that more variables should be considered. Presumably, one of them is the peptide ability to self-associate what was determined in this study. Only *T*_max_ value of temporin A analog is higher than that of the parent molecule (Table 5). This information indicates that r-temporin A exhibited an increased tendency to self-associate. According to the previous statement that self-association facilitates peptide elution in RP-HPLC, the observed relation became more apparent. However, the same cannot be stated about pexiganan. Moreover, CD results (Table 3) indicate that only retro analogs of pexiganan and temporin A exhibited smaller helicity fractions in both solutions, SDS and DPC. Importantly, the enhanced peptide helicity is likely to significantly contribute to longer peptide retention time (Blondelle et al. 1995). Presumably, one of the reasons of the earlier retention of r-pexiganan and r-temporin A is the decreased helicity fraction. Doig and Baldwin (1995) investigated N- and C-capping preferences in α-helical peptides. The free energies of N- and C-capping indicated that the latter has a little impact on α-helix stability. In contrast to N-terminal glycine present in pexiganan, N-terminal lysine in its retro analog probably exerts a significant helix destabilizing effect. Considering all the facts about r-pexiganan, it can be suggested that its antimicrobial activity against Gram-negative strains is not closely related to hydrophobicity and helicity in contrast to those of other helical AMPs.

## Conclusions

All of the studied compounds exhibited antimicrobial activity against most of the tested strains; although, essentially different. Most of the tested peptide and retro peptide pairs exhibited a lower activity of original molecules, except r-omiganan. Additionally, omiganan differs from the remaining retro analogs by its higher hemolytic activity compared to the parent peptide. This study focuses partially on the interplay between peptide molecules. Thanks to chromatographic analysis over the wide temperature range it was possible to assess peptides’ tendency to self-association which was as a rule different between peptides in pairs. Being aware of the influence of self-association on retention time, it was expedient to apply the temperature gradient, this providing a reliable point of view on the peptides’ hydrophobicity. Moreover, the tendency to self-association and helicity fraction (fH) in SDS seems to be correlated. The normalized RP-HPLC—temperature profiles of citropin 1.1 and aurein 1.2, revealed an enhanced tendency to self-association than that of their retro analogs. Considering their mode of action, this tendency is likely to be involved in antimicrobial activity. It seems that it is possible to predict a substantial effect of sequence reversion on peptide hydrophobicity using side-chain hydrophobicity coefficients and thus to expect particular change in antimicrobial activity—enhance when hydrophobicity is increasing. However, this approach may fail if the difference is not high enough as it was found in this study (pexiganan and temporin A). Our results indicate that it is possible to reduce the number of those fails. If calculations based on hydrophobicity coefficients indicate that retro analog will be substantially more hydrophilic, there is a high risk that it will also be less antimicrobial active. Nevertheless, this approach does not allow to predict all changes in the biological activity, e.g., increase in activity against particular strain in antimicrobial assay (r-pexiganan and *K. pneumoniae*). Hypothetically, to increase the chance of obtaining more active compounds, retro analogs should be synthesized after initial calculations. However, this approach needs further studies to verify its correctness, range of applicability, and to determine guidelines to follow. Limited scope of this work does not allow us to draw more general statements but only to indicate which aspects should be taken into account. In conclusion, the retro analog concept applied to antimicrobial peptides presumably may contribute to development of more active peptides.

## Abbreviations

ACN: Acetonitrile
AMPs: Antimicrobial peptides
ATCC: American Type Culture Collection
CD: Circular dichroism
CLSI: Clinical and Laboratory Standards Institute
DCM: Dichloromethane
DIC: *N,N′*-diisopropylcarbodiimide
DMF: *N,N*-dimethylformamide
DPC: Dodecylphosphocholine
EDTA: Ethylenediaminetetraacetic acid
ESI–MS: Electrospray-ionization mass spectrometry
fH: Helicity fraction
Fmoc: 9-Fluorenylmethoxycarbonyl group
MIC: Minimal inhibitory concentration
MRME: Mean residue molar ellipticity
PBS: Phosphate-buffer saline
PCM: Polish Collection of Microorganisms
RBCs: Red blood cells
RP-HPLC: Reverse-phase high-performance liquid chromatography
SDS: Sodium dodecyl sulfate
SPPS: Solid-phase peptide synthesis
TFA: Trifluoroacetic acid
TFE: Trifluoroethanol
TIS: Triisopropylsilane
*T*_max_: A maximum on a normalized plot of the change in retention time at different temperatures
WHO: World Health Organization

## References

[CR1] Ambroggio EE, Separovic F, Bowie JH (2005). Direct visualization of membrane leakage induced by the antibiotic peptides: maculatin, citropin, and aurein. Biophys J.

[CR2] Ando S, Mitsuyasu K, Soeda Y (2010). Structure-activity relationship of indolicidin, a Trp-rich antibacterial peptide. J Pept Sci.

[CR3] Andreu D, Ubach J, Boman A (1992). Shortened cecropin A-melittin hybrids. Significant size reduction retains potent antibiotic activity. FEBS Lett.

[CR4] Apponyi MA, Pukala TL, Brinkworth CS (2004). Host-defence peptides of Australian anurans: structure, mechanism of action and evolutionary significance. Peptides.

[CR5] Avrahami D, Shai Y (2004). A new group of antifungal and antibacterial lipopeptides derived from non-membrane active peptides conjugated to palmitic acid. J Biol Chem.

[CR6] Baranska-Rybak W, Cirioni O, Dawgul M (2011). Activity of antimicrobial peptides and conventional antibiotics against superantigen positive *Staphylococcus aureus* isolated from the patients with neoplastic and inflammatory erythrodermia. Chemother Res Pract.

[CR7] Bhargava K, Feix JB (2004). Membrane binding, structure, and localization of cecropin-mellitin hybrid peptides: a site-directed spin-labeling study. Biophys J.

[CR8] Blondelle SE, Ostresh JM, Houghten RA, Pérez-Payá E (1995). Induced conformational states of amphipathic peptides in aqueous/lipid environments. Biophys J.

[CR9] Boman HG (2003). Antibacterial peptides: basic facts and emerging concepts. J Intern Med.

[CR10] Cinelli S, Spinozzi F, Itri R (2001). Structural characterization of the pH-denatured states of ferricytochrome-c by synchrotron small angle X-ray scattering. Biophys J.

[CR11] Clinical and Laboratory Standards Institute (CLSI) (2002) Reference method for broth dilution antifungal susceptibility testing of yeasts. Approved Standards-Second Edition, in CLSI document M27-2A 2002, CLSI Pennsylvania, USA

[CR12] Clinical and Laboratory Standards Institute (CLSI) (2012) Methods for dilution antimicrobial susceptibility tests f or bacteria that grow aerobically. Approved Standard—Ninth Edition

[CR13] Cornish J, Callon KE, Lin CQ (1999). Trifluoroacetate, a contaminant in purified proteins, inhibits proliferation of osteoblasts and chondrocytes. Am J Physiol.

[CR14] Dennison SR, Harris F, Phoenix DA (2007). The interactions of aurein 1.2 with cancer cell membranes. Biophys Chem.

[CR15] Doig AJ, Baldwin RL (1995). N- and C-capping preferences for all 20 amino acids in α-helical peptides. Protein Sci.

[CR16] Faccone D, Veliz O, Corso A (2014). Antimicrobial activity of de novo designed cationic peptides against multi-resistant clinical isolates. Eur J Med Chem.

[CR17] Fields GB, Noble RL (1990). Solid phase peptide synthesis utilizing 9-fluorenylmethoxycarbonyl amino acids. Int J Pept Protein Res.

[CR18] Flamm RK, Rhomberg PR, Simpson KM (2015). In vitro spectrum of pexiganan activity when tested against pathogens from diabetic foot infections and with selected resistance mechanisms. Antimicrob Agents Chemother.

[CR19] Fosgerau K, Hoffmann T (2015). Peptide therapeutics: current status and future directions. Drug Discov Today.

[CR20] Ge Y, MacDonald D, Henry MM (1999). In vitro susceptibility to pexiganan of bacteria isolated from infected diabetic foot ulcers. Diagn Microbiol Infect Dis.

[CR21] Ge Y, MacDonald DL, Holroyd KJ (1999). In vitro antibacterial properties of pexiganan, an analog of magainin. Antimicrob Agents Chemother.

[CR22] Giacometti A, Cirioni O, Kamysz W (2005). In vitro activity of citropin 1.1 alone and in combination with clinically used antimicrobial agents against *Rhodococcus equi*. J Antimicrob Chemother.

[CR23] Giacometti A, Cirioni O, Kamysz W (2005). In vitro activity and killing effect of citropin 1.1 against gram-positive pathogens causing skin and soft tissue infections. Antimicrob Agents Chemother.

[CR24] Giacometti A, Cirioni O, Riva A (2007). In vitro activity of aurein 1.2 alone and in combination with antibiotics against gram-positive nosocomial cocci. Antimicrob Agents Chemother.

[CR25] Girmenia C, Serrao A, Canichella M (2016). Epidemiology of carbapenem resistant *Klebsiella pneumoniae* infections in Mediterranean Countries. Mediterr J Hematol Infect Dis.

[CR26] Gopal R, Kim YJ, Seo CH (2011). Reversed sequence enhances antimicrobial activity of a synthetic peptide. J Pept Sci.

[CR27] Hancock RE, Lehrer R (1998). Cationic peptides: a new source of antibiotics. Trends Biotechnol.

[CR28] ICH (2005) Validation of analytical procedures : text and methodology Q2 (R1). In: International Conference on Harmonization, pp 1–13

[CR29] Kamysz W, Nadolski P (2005). Antibacterial activity of peptides from amphibians skin (PL: Przeciwbakteryjna aktywność peptydów ze skóry płazów). Acad Med Gedan.

[CR30] Kamysz W, Okrój M, Łukasiak J (2003). Novel properties of antimicrobial peptides. Acta Biochim Pol.

[CR31] Kamysz W, Mickiewicz B, Rodziewicz-Motowidło S (2006). Temporin A and its retro-analogues: synthesis, conformational analysis and antimicrobial activities. J Pept Sci.

[CR32] Kamysz W, Mickiewicz B, Greber K, Rodziewicz-Motowidło S (2007). Conformational solution studies of the anti-microbial temporin A retro-analogues by using NMR spectroscopy. J Pept Sci.

[CR33] Kaspar AA, Reichert JM (2013). Future directions for peptide therapeutics development. Drug Discov Today.

[CR34] Kim S, Kim SS, Lee BJ (2005). Correlation between the activities of alpha-helical antimicrobial peptides and hydrophobicities represented as RP HPLC retention times. Peptides.

[CR35] Lee DL, Mant CT, Hodges RS (2003). A novel method to measure self-association of small amphipathic molecules: temperature profiling in reversed-phase chromatography. J Biol Chem.

[CR36] Lopez-Medina E, Fan D, Coughlin LA (2015). *Candida albicans* inhibits *Pseudomonas aeruginosa* virulence through suppression of pyochelin and pyoverdine biosynthesis. PLoS Pathog.

[CR37] Lorenzón EN, Piccoli JP, Cilli EM (2014). Interaction between the antimicrobial peptide aurein 1.2 dimer and mannans. Amino Acids.

[CR38] Mangoni ML, Rinaldi AC, Di Giulio A (2000). Structure–function relationships of temporins, small antimicrobialpeptides from amphibian skin. Eur J Biochem.

[CR39] Mansour SC, Pena OM, Hancock RE (2014). Host defense peptides: front-line immunomodulators. Trends Immunol.

[CR40] Merrifield RB, Juvvadi P, Andreu D (1995). Retro and retroenantio analogs of cecropin–melittin hybrids. Proc Natl Acad Sci USA.

[CR41] Nagpal S, Kaur KJ, Jain D, Salunke DM (2002). Plasticity in structure and interactions is critical for the action of indolicidin, an antibacterial peptide of innate immune origin. Protein Sci.

[CR42] Oh H, Hedberg M, Wade D, Edlund C (2000). Activities of synthetic hybrid peptides against anaerobic bacteria: aspects of methodology and stability. Antimicrob Agents Chemother.

[CR43] Pini A, Lozzi L, Bernini A (2012). Efficacy and toxicity of the antimicrobial peptide M33 produced with different counter-ions. Amino Acids.

[CR44] Porcelli F, Buck-Koehntop BA, Thennarasu S (2006). Structures of the dimeric and monomeric variants of magainin antimicrobial peptides (MSI-78 and MSI-594) in micelles and bilayers, determined by NMR spectroscopy. Biochemistry.

[CR45] Rao T, Ruiz-Gómez G, Hill TA (2013). Truncated and helix-constrained peptides with high affinity and specificity for the cFos coiled-coil of AP-1. PLoS One.

[CR46] Rinaldi AC (2002). Antimicrobial peptides from amphibian skin: an expanding scenario: commentary. Curr Opin Chem Biol.

[CR47] Rozek A, Friedrich CL, Hancock REW (2000). Structure of the bovine antimicrobial peptide indolicidin bound to dodecylphosphocholine and sodium dodecyl sulfate micelles. Biochemistry.

[CR48] Rozek T, Wegener KL, Bowie JH (2000). The antibiotic and anticancer active aurein peptides from the Australian Bell Frogs *Litoria aurea* and *Litoria raniformis*. Eur J Biochem.

[CR49] Sader HS, Fedler KA, Rennie RP (2004). Omiganan pentahydrochloride (MBI 226), a topical 12-amino-acid cationic peptide: spectrum of antimicrobial activity and measurements of bactericidal activity. Antimicrob Agents Chemother.

[CR50] Saravanan R, Joshi M, Mohanram H (2013). NMR structure of temporin-1 Ta in lipopolysaccharide micelles: mechanistic insight into inactivation by outer membrane. PLoS One.

[CR51] Schauber J, Gallo RL (2007). Expanding the roles of antimicrobial peptides in skin: alarming and arming keratinocytes. J Invest Dermatol.

[CR52] Sereda TJ, Mant CT, Quinn AM, Hodges RS (1993). Effect of the α-amino group on peptide retention behaviour in reversed-phase chromatography determination of the p*K*a values of the α-amino group of 19 different N-terminal amino acid residues. J Chromatogr A.

[CR53] Shahmiri M, Enciso M, Mechler A (2015). Controls and constrains of the membrane disrupting action of aurein 1.2. Sci Rep.

[CR54] Shanmugam G, Polavarapu PL, Gopinath D, Jayakumar R (2005). The structure of antimicrobial pexiganan peptide in solution probed by Fourier transform infrared absorption, vibrational circular dichroism, and electronic circular dichroism spectroscopy. Biopolymers.

[CR55] Sikorska E, Greber K, Rodziewicz-Motowidło S (2009). Synthesis and antimicrobial activity of truncated fragments and analogs of citropin 1.1: the solution structure of the SDS micelle-bound citropin-like peptides. J Struct Biol.

[CR56] Simmaco M, Mignogna G, Canofeni S (1996). Temporins, antimicrobial peptides from the European red frog *Rana temporaria*. Eur J Biochem.

[CR57] Smolarczyk R, Cichoń T, Kamysz W (2010). Anticancer effects of CAMEL peptide. Lab Investig.

[CR58] Subbalakshmi C, Nagaraj R, Sitaram N (2001). Biological activities of retro and diastereo analogs of a 13-residue peptide with antimicrobial and hemolytic activities. J Pept Res.

[CR59] Tripet B, Cepeniene D, Kovacs JM (2007). Requirements for prediction of peptide retention time in reversed-phase high-performance liquid chromatography: hydrophilicity/hydrophobicity of side-chains at the N- and C-termini of peptides are dramatically affected by the end-groups and location. J Chromatogr A.

[CR60] Wabnitz PA, Bowie JH, Wallace JC, Tyler MJ (1999). The citropin peptides from the skin glands of the Australian Blue Mountains tree frog *Litoria citropa*. Part 2: sequence determination using electrospray mass spectrometry. Rapid Commun Mass Spectrom.

[CR61] Wada K, Mizuno T, Oku J-I, Tanaka T (2003). pH-induced conformational change in an alpha-helical coiled-coil is controlled by His residues in the hydrophobic core. Protein Pept Lett.

[CR62] Wegener KL, Wabnitz PA, Carver JA (1999). Host defence peptides from the skin glands of the Australian Blue Mountains tree-frog *Litoria citropa*. Solution structure of the antibacterial peptide citropin 1.1. Eur J Biochem.

[CR63] World Health Organization (2014) Antimicrobial resistance: global report on surveillance. HO Press, World Health Organization, Switzerland

[CR64] Zasloff M (2002). Antimicrobial peptides of multicellular organisms. Nature.

